# Prostaglandin E2 promotes embryonic vascular development and maturation in zebrafish

**DOI:** 10.1242/bio.039768

**Published:** 2019-04-15

**Authors:** Kingsley Chukwunonso Ugwuagbo, Sujit Maiti, Ahmed Omar, Stephanie Hunter, Braydon Nault, Caleb Northam, Mousumi Majumder

**Affiliations:** Department of Biology, Brandon University, Brandon, Manitoba R7A 6A9, Canada

**Keywords:** Zebrafish, Prostaglandin E2 (PGE2), Vascular development and maturation, Angiogenesis

## Abstract

Prostaglandin (PG)-E2 is essential for growth and development of vertebrates. PGE2 binds to G-coupled receptors to regulate embryonic stem cell differentiation and maintains tissue homeostasis. Overproduction of PGE2 by breast tumor cells promotes aggressive breast cancer phenotypes and tumor-associated lymphangiogenesis. In this study, we investigated novel roles of PGE2 in early embryonic vascular development and maturation with the microinjection of PGE2 in fertilized zebrafish (*Danio rerio*) eggs. We injected Texas Red dextran to trace vascular development. Embryos injected with the solvent of PGE2 served as vehicle. Distinct developmental changes were noted from 28–96 h post fertilization (hpf), showing an increase in embryonic tail flicks, pigmentation, growth, hatching and larval movement post-hatching in the PGE2-injected group compared to the vehicle. We recorded a significant increase in trunk vascular fluorescence and maturation of vascular anatomy, embryo heartbeat and blood vessel formation in the PGE2 injected group. At 96 hpf, all larvae were euthanized to measure vascular marker mRNA expression. We observed a significant increase in the expression of stem cell markers *efnb2a*, *ephb4a*, angiogenesis markers *vegfa*, *kdrl*, *etv2* and lymphangiogenesis marker *prox1* in the PGE2-group compared to the vehicle. This study shows the novel roles of PGE2 in promoting embryonic vascular maturation and angiogenesis in zebrafish.

This article has an associated First Person interview with the first author of the paper.

## Abstract (precis)

**Summary:** Overproduction of PGE2 causes inflammation and cancer and its blockage results in serious physiological consequences. This study investigated the novel roles of PGE2 in early vascular development of zebrafish.

## INTRODUCTION

Prostaglandin (PG) E-2 is a prostanoid, which is endogenously synthesized from the arachidonic acid of vertebrate cell membranes by an enzyme, cyclooxygenase (COX)-2. PGE2 binds to four different G-coupled receptors (EPs), EP 1–4, which have various cellular functions (Sugimoto and Narumiya, 2007). EP2 and EP4 receptors share the cAMP/PKA pathway; however, EP4 additionally signals through the phosphatidylinositol 3-kinase (PI3K)/AKT pathway (Fujino et al., 2003). In vertebrates, PGE2 plays major physiological roles in embryonic development by regulating the homeostatic balance of hematopoietic stem cells (HSC) during early embryonic growth. The role of PGE synthase (*Ptges*) in embryonic differentiation and growth in zebrafish was well investigated by Cha et al. (2006). They showed that knockdown of *Ptges* in the zebrafish embryo completely abrogated early cell differentiation and cell polarization, which was retrieved with PGE2 addition, as embryos could recover all phenotypes. They have also shown that PGE2 regulates zebrafish growth via EP4/PI3K/Akt pathways (Cha et al., 2006). Supporting this, North et al. (2007) showed that PGE2 regulates the differentiation of embryonic stem cells. This study showed that treatment with chemicals that enhance PGE2 synthesis induced HSC and in contrast, chemicals, which block prostaglandin synthesis, decreased stem cell numbers (North et al., 2007).

PGE2 induces breast cancer stem-like cells (SLCs) via the upregulation of stem cells marker (NANOG and SOX2) in tumors and by stimulation of *NOTCH* and *Wnt* genes expression (Majumder et al., 2016). With a COX-2 inhibitor (COX2-I) and specific EP4 antagonist (EP4A) treatment, we could abrogate COX-2/PGE2 induced SLCs in breast cancer. In addition, PI3K/Akt inhibitors also abrogated PGE2 induced *NOTCH* and *WNT* genes expression in human breast cancer. Therefore, we have established that COX-2 and PGE2 induce human breast SLCs and were regulated by EP4/PI3K/Akt/NOTCH/WNT pathways (Majumder et al., 2016). However, we never tested the roles of PGE2 in vertebrate vascular development.

Zebrafish are widely used as vertebrate models for pathophysiological studies (North et al., 2007; Zon and Peterson, 2005). The transparency of the zebrafish identifies it as a good model for the investigation of vascular development. Moreover, zebrafish mutually share many structural, functional and molecular features with other vertebrates and are perfect models for xenotransplantation due to their inability to reject graft within 48 hpf (Benyumov et al., 2012; Jung et al., 2017; Mulligan and Weinstein, 2014). In vertebrates, lymphangiogenesis begins from HSC fate determination, through several differentiation steps to develop arterial and venous progenitors (Nicenboim et al., 2015). The genetic interactions involving HSC homeostasis and replenishment in zebrafish is regulated by PGE2 through the Wnt pathway (Goessling et al., 2009). Similarly, cardiac muscle development and pigmentation in zebrafish was shown to be regulated through Wnt signaling (Dohn and Waxman, 2012; Vibert et al., 2017). In animal models, cell lineage and vascular differentiation stages could be monitored with Texas Red low-molecular-weight dextran, a widely used fluorescent dye for tracing vascular lineages in vertebrate development (Zhao et al., 2011). There is no comprehensive report on the roles of PGE2 in zebrafish embryonic angiogenesis and lymphangiogenesis. Therefore in this study, we investigated the effects of PGE2 on zebrafish embryonic vascular development and maturation.

The process of vascular development and maturation principally involves the interaction of vascular endothelial growth factors (*vegf*)s with their cognate receptors (*vegfr*)s in zebrafish. VEGF/VEGFR interaction plays a critical role in the formation and modification of vascular network during embryonic development in vertebrates. In zebrafish, primarily *vegfa* and *vegfd* directly interact with *kdrl* (equivalent to *VEGFR2* in human) to regulate angiogenesis and lymphangiogenesis during early embryonic growth (Covassin et al., 2006; Bahary et al., 2007; Bower et al., 2017). Alternatively, angiogenesis is partly regulated by *vegfa* receptor *flt1* (equivalent to *VEGFR1* in humans), tested in mouse endothelial cell models (Nesmith et al., 2017). In zebrafish, angiogenesis involves a coordinated regulation of *kdrl* and *flt4* (equivalent to *VEGFR3* in humans) receptors, partly controlled by Erk and Notch signaling (Phng and Gerhardt, 2009). Expression of NOTCH transmembrane ligand *efnb2a* and its cognate receptor *ephb4a* on vascular endothelial cells and blood vessels selectively promotes cardiovascular development and angiogenesis in mouse embryos (Gerety, et al., 1999; Chen et al., 2015). However, Krueger et al. (2011) showed that *flt1* negatively regulates *efnb2a* during early angiogenic sprouting and erythropoiesis in zebrafish (Krueger et al., 2011). Furthermore, the roles of another vascular transcription factor *etv2* and its receptor G protein gamma-2 in VEGF-mediated angiogenesis and lymphangiogenesis in vertebrates remains unclear (Leung et al., 2006; Gomez et al., 2009; Davis et al., 2018). Roles of PGE2 in vascular marker expression have never been tested in zebrafish.

Previously, we have shown that in mouse breast cancer cell lines COX-2 induces PGE2 synthesis, which in turn induces tumor-associated angiogenesis and lymphangiogenesis via overproduction of VEGF-C and VEGF-D. This was regulated by EP4/PI3K/Akt pathway, and selective COX-2I and EP4A could inhibit tumor associated angiogenesis and metastasis in a mouse model (Xin et al., 2012; Majumder et al., 2014; Lala et al., 2018). Furthermore, using rat lymphatic mesenteric lymphatic endothelial cells (RMLEC)s we showed that PGE2 induced lymphangiogenesis *in vitro* could be abrogated with COX2-I and EP4A (Nandi et al., 2017). In human breast cancer we have also shown that PGE2 induces cancer cell migration, invasion and tumor-associated angiogenesis and lymphangiogenesis via upregulation of the EP4/PI3K/AKT pathways. Each of these phenotypes could be abrogated with a specific COX-2I and an EP4A (Majumder et al., 2016, 2018). Non-steroidal anti-inflammatory drugs (NSAIDs) have emerged as powerful COX-2 inhibitors, which inhibit the synthesis of prostaglandins, hence are commonly used as pain medication (Ricciotti and FitzGerald, 2011). However, chronic consumption of NSAIDs by North Americans resulted in severe side effects like gastrointestinal ulcers, perforation, bleeding, cardiac strokes, myocardial infarction, hypertension and renal dysfunction (Harirforoosh et al., 2013; Pai et al., 2018). Most of these effects might be due to blockage of protective physiological functions of PGE2 in humans (Guo et al., 2012; Przygodzki et al., 2015). So, in this article, we tested the physiological roles of PGE2 during early embryonic vascular growth and maturation using zebrafish as an *in vivo* model.

## RESULTS

### PGE2 induces early embryonic movement and tail flicks

**Fig. 1.**
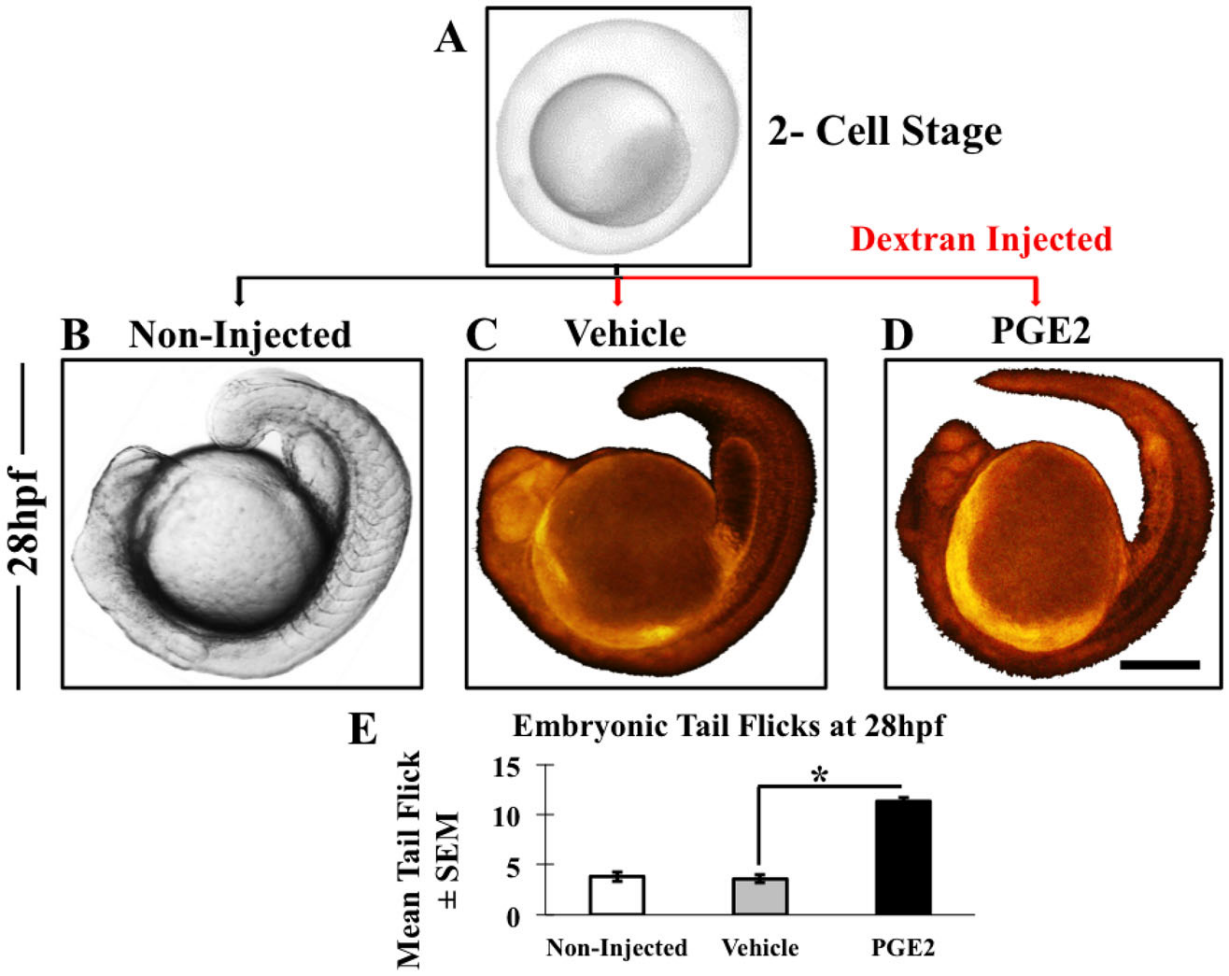
**Microinjection and embryonic movement at 28 hpf.** (A) We collected zebrafish eggs at the two-cell stage and a few non-injected embryos were kept as a reference (B). Only vehicle (C) and PGE2 (D) embryos were fluorescently labeled due to dextran injection. We monitored post-injection growth of embryos with both stereo and fluorescent microscopes and observed an increase in embryonic tail flicks in the PGE2 group (shown in Movie 1A-C). (E) From each biological replicate we selected some embryos to measure tail flicks. Chart showing the mean of embryonic tail flicks of non-injected (*n*=17), vehicle (*n*=26) and PGE2 (*n*=26) injected embryos±s.e.m. We conducted an unpaired *t*-test and results show a significant increase in tail flicks in PGE2 injected group compared to the vehicle with a two-tailed unpaired *t*-test, *P=0.0001. Scale bar: 5 μm in A; 25 μm in B,C,D.

To investigate an early embryonic phenotype in zebrafish, we injected fertilized eggs at the 2-cell stage with either PGE2 (4 μM) or vehicle of PGE2 (0.13% BSA) along with Texas-Red dextran (2 μM) (Fig. 1A–D). Some non-injected embryos groups served as the control. Almost 99% of embryos in PGE2 and vehicle showed fluorescence in all replicates (data not presented), and the embryos without fluorescence were excluded from the study.

We measured the embryonic tail-flick frequency at 28 h post fertilization (hpf) in selective embryos. Embryonic tail flicks for the non-injected (*n*=17) (Fig. 1B; Movie 1A), vehicle injected (*n*=26) (Fig. 1C; Movie 1B) and PGE2 injected (*n*=26) (Fig. 1D; Movie 1C) embryos were recorded and quantified by counting the number of spontaneous tail movements of each embryo for a 30 s exposure. We conducted an unpaired *t*-test comparing the mean and standard error of the mean (s.e.m.) in PGE2 and vehicle groups and results showed a significant increase in embryonic tail flicks in PGE2 group *P*=0.004 (Fig. 1E). These data suggest that PGE2 enhanced early embryonic movement at 28 hpf.

### PGE2 increases embryonic pigmentation and growth

**Fig. 2.**
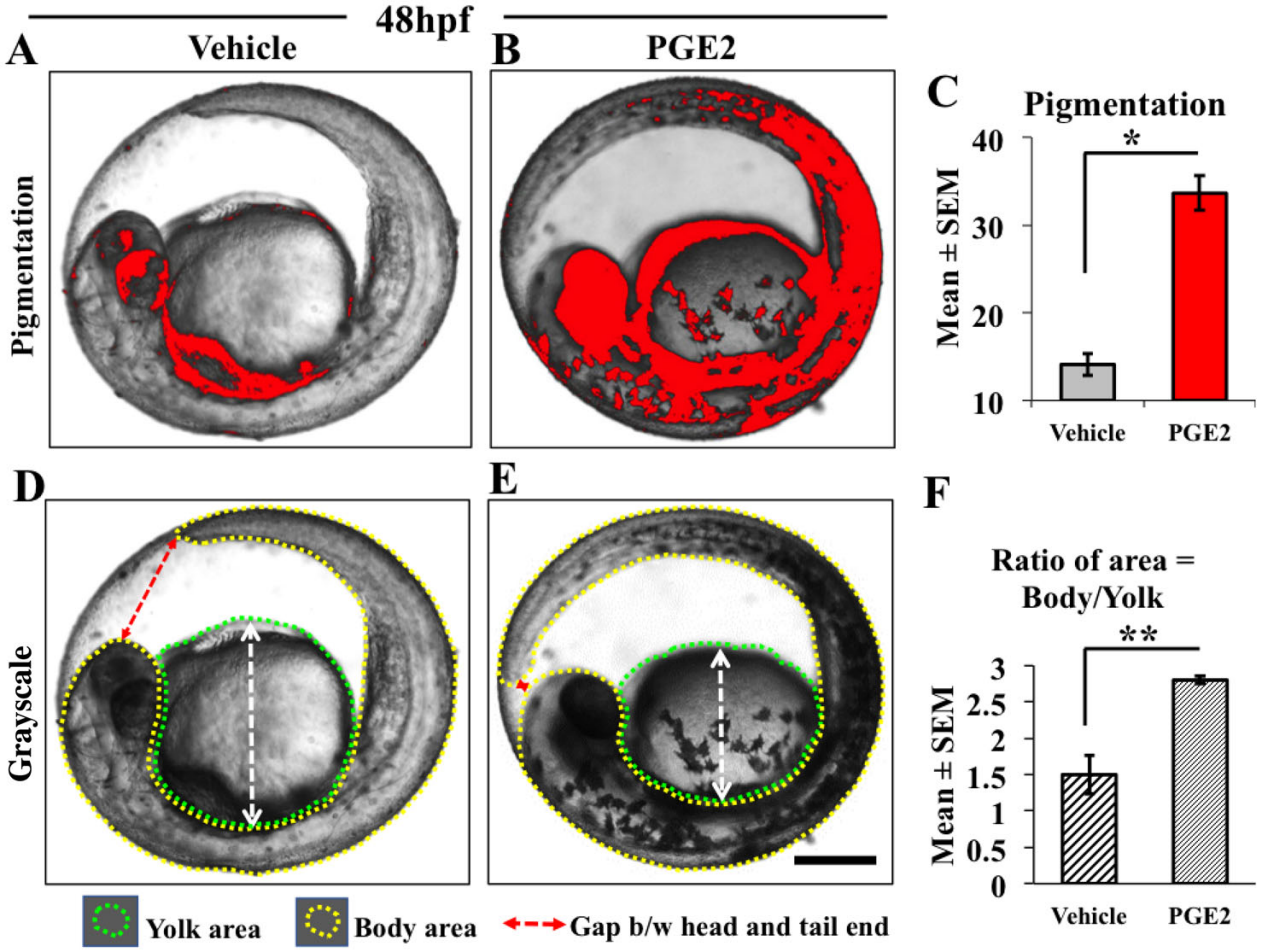
**PGE2 increases zebrafish embryonic development and pigmentation at 48 hpf**. For the measurement of pigmentation, the gray-scaled images from the stereomicroscope were converted to red-colored scale using ImageJ. The red color intensity of the pigmented areas in the vehicle (A) was used as a threshold to measure PGE2-induced pigmentation (B). We selected a few embryos from three biological replicates for this quantification. (C) Data are represented as mean of embryonic pigmentation for PGE2 (*n*=10) and vehicle (*n*=11) groups±s.e.m. An unpaired *t*-test comparing means showed a significant upregulation of embryonic pigmentation in PGE2 group with a two-tailed unpaired *t*-test, *P=0.0001. (E) The PGE2-treated fish is larger with the tail almost reaching the head in the embryonic sac (dotted red line with arrow) compared to the vehicle (D). We measured the areas of the fish body (denoted with yellow dotted line) and yolk sac (denoted with green dotted line) and calculated the ratio of the areas as body/yolk. (F) The chart shows a very significant growth difference between the PGE2 and vehicle groups with a two-tailed unpaired *t*-test, **P=0.0086. Scale bar: 10 μm.

Along with improved embryonic movement, we also observed a visible change in embryonic pigmentation. So, we measured the intensity of embryonic pigmentation at 48 hpf and collected data from the selective vehicle (*n*=11) (Fig. 2A) and PGE2 injected (*n*=10) (Fig. 2B) embryos from all replicates. We used vehicle pigmentation as a threshold to measure PGE2 pigmentation (Fig. 2C) using ImageJ. Quantification showed a significant increase in the pigmentation in the PGE2 injected embryos compared to the vehicle at 48 hpf, *P*=0.014 (Fig. 2C), data for non-injected are not presented. Increase in the pigmentation is an indicator of progressive development (Kimmel et al., 1995).

Additionally, we noticed PGE2 embryos growing faster and getting bigger, so we measured yolk to larval area ratio (larval curvature within the embryo). We observed a decrease in yolk sac areas with a corresponding increase of the larval (head to tail) area in PGE2 embryos (Fig. 2D) compared to vehicle (Fig. 2E) with a significant result, *P*=0.0086 (Fig. 2F). No difference was observed in the yolk to larval area ratios between the non-injected and the vehicle (data not presented). These data further suggest that PGE2 promotes embryonic growth and maturation of zebrafish embryos.

### PGE2 promotes early hatching and movement of embryos at 50 hpf

**Fig. 3.**
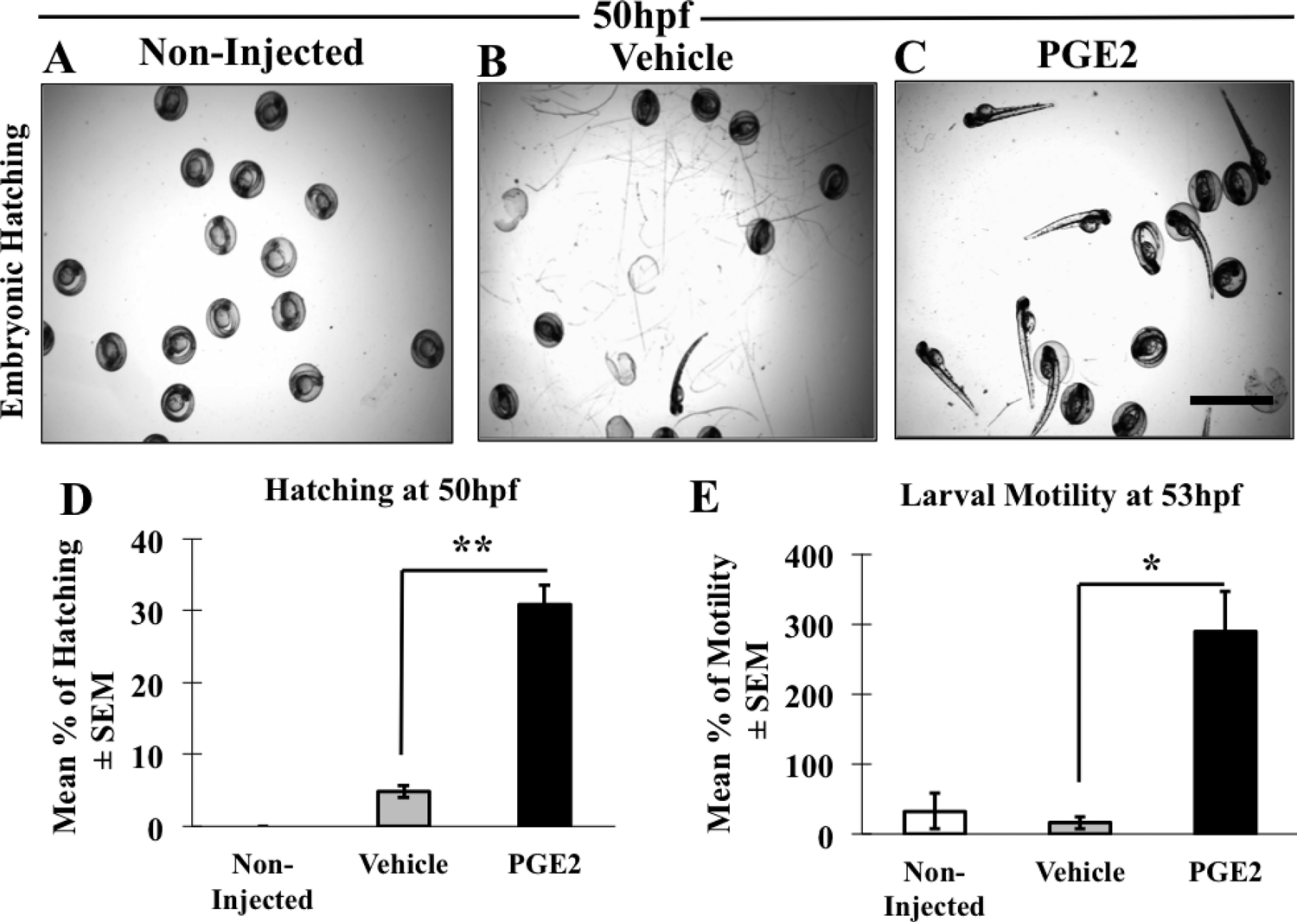
**PGE2 promotes early hatching of zebrafish at 50 hpf.** (A–C) Representative images of hatched embryos in non-injected (A), vehicle (B) and PGE2 injected (C) groups. Scale bar: 10 μm. We analyzed *n*=90 non-injected, *n*=61 vehicle and *n*=90 PGE2 embryos. (D) Data are presented as the mean of percentages (hatched/total number of eggs) of hatched embryos±s.e.m. Hatched embryo numbers were significantly high in PGE2 (33%) compared with the vehicle (5%), with a two-tailed unpaired *t*-test, **P=0.0001. (E) We also recorded the larval movements as a measure of swimming activity post-hatching in all three groups (shown in Movie 2A-C) at 53 hpf. Data presented as the mean of larval movements of hatched embryos [non-injected (*n*=14), vehicle (*n*=31) and PGE2 (*n*=35)]±s.e.m. We conducted an unpaired *t*-test showing significantly higher motility in PGE2 group compared to the vehicle with a two-tailed unpaired *t*-test, **P=0.0008.

To further investigate advanced morphological development due to PGE2, we monitored hatching time of non-injected (*n*=90) (Fig. 3A), vehicle (*n*=61) (Fig. 3B) and PGE2 (*n*=90) (Fig. 3C) embryos in all three replicates. We observed a significant increase in the hatching rate at 50 hpf in PGE2 group compared to the vehicle, *P*=0.0001. At this time point, no embryo hatched in the non-injected embryos (Fig. 3A). Overall, only 5% of the vehicle and approximately 40% of the PGE2 embryos had hatched at 50 hpf (Fig. 3D) including all replicates. The non-injected embryos hatched at 53 h.

Usually zebrafish embryos follow a resting phase immediately after hatching (Kimmel et al., 1995), which is characterized by physical inactivity. To further associate PGE2-induced early larval development we recorded the swimming activity of zebrafish larvae from 50–53 hpf, immediately after hatching. Embryos were resting post-hatching in the non-injected (Movie 2A) and the vehicle (Movie 2B) groups. However, larvae in PGE2 group were very active immediately after hatching at 50 hpf (Movie 2C). We quantified any movement of the hatched larvae (swimming activity) at 53 hpf in all three groups for 30 s. A significant increase in the movement of embryos was recorded in the PGE2 group while only a few hatched larvae showed any activity in the vehicle group, with a significant result, *P*=0.0001 (Fig. 3E). The larvae were more actively swimming in the PGE2 group as a result of an advanced stage of growth (Kimmel et al., 1995) due to PGE2 stimulation. No difference was seen in larval movements between non-injected and vehicle larvae after hatching. The observed early hatching and enhanced swimming activity of the larvae due to PGE2 suggests that PGE2 promotes improved physiological activity in zebrafish, which might need an active vascular system.

### PGE2 induced early vascular maturation and increased heartbeat in zebrafish

**Fig. 4.**
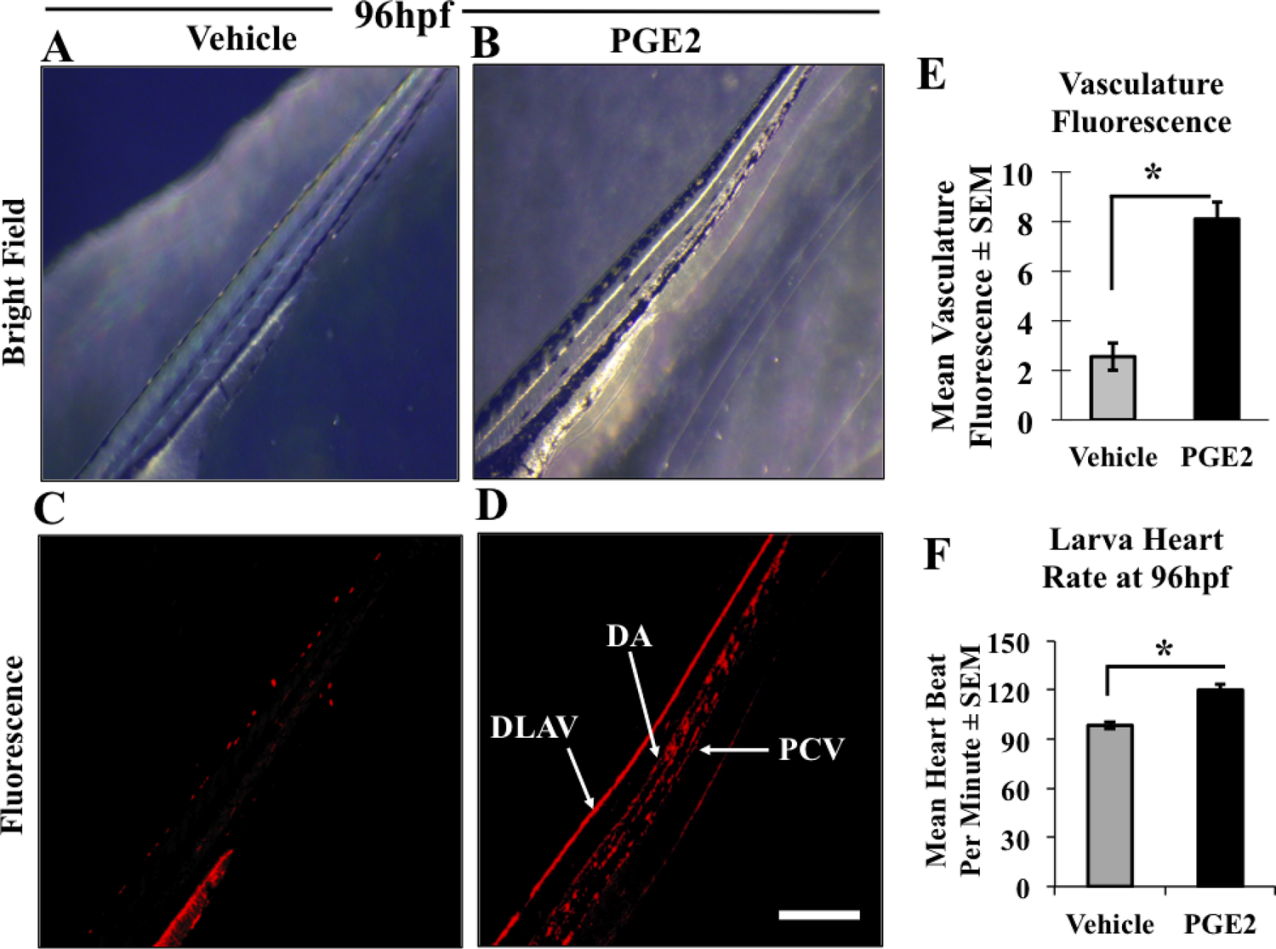
**PGE2 induced vascular maturation and increased heart rate in zebrafish.** (A,B) Gray-scaled images of trunk vasculature of developing zebrafish in both vehicle and PGE2 groups at 96 hpf. Both vehicle and PGE2 groups were microinjected with Texas-Red dextran dye and grown under the same conditions, with vasculature formation captured from 53 hpf to 96 hpf, data presented only for 96 hpf. (C,D) Fluorescence images of trunk vasculature in the vehicle and PGE2 larvae. Vehicle color was considered as a threshold to measure fluorescence of the PGE2 using ImageJ. (E) The chart showing the mean of trunk vascular fluorescence measured for both vehicle (*n*=9) and PGE2 (*n*=9) larvae±s.e.m. The PGE2 group showed a significant (*P=0.0001) increase in fluorescence compared to the vehicle group. The PGE2-injected larvae also showed a clear formation of the mature vasculature with dorsal longitudinal anastomosing vessel (DLAV), intersegmental arteries (ISA), dorsal aorta (DA) and posterior cardinal vein (PCV) formation while the vehicle remained premature. (F) The chart shows the mean heart rate of selective embryos from three biological replicates (*n*=12 for vehicles and *n*=15 for PGE2)±s.e.m. An unpaired *t*-test showed a significantly high heart rate in the PGE2 group compared to the vehicle group larvae at 72 hpf, with a two-tailed unpaired *t*-test, **P=0.0221. Video data for non-injected presented in Movie 3A, vehicle injected in Movie 3B and PGE2 injected in Movie 3C. Scale bar: 10 μm.

Due to the increased embryonic growth and early hatching observed in the PGE2 group, we hypothesized that to support the physiologic activity of hatched larvae they need a functional vascular supply. Therefore, we sought to characterize the role of PGE2 in the vascular development in zebrafish. We injected Texas-Red dextran, a red fluorescent dye that can trace cell lineages *in vivo*. We monitored fluorescently labeled cell lineages in the hatched embryos in both vehicle and PGE2 groups from 50 hpf to 96 hpf, with a fluorescent microscope. A more developed vasculature in the trunk areas of the developing larvae in the PGE2 group (Fig. 4B) compared to the vehicle (Fig. 4A) was recorded. Specifically, fluorescent images showing the trunk vascular fluorescence are illustrated as a more developed dorsal aorta (DA), posterior cardinal vein (PCV) and dorsal longitudinal anastomotic vessel (DLAV) in the PGE2 (Fig. 4D) compared to the vehicle (Fig. 4C). We measured total fluorescent intensity in selective embryos (*n*=9 for vehicle and *n*=9 for PGE2) with ImageJ and the quantification of images at 96 hpf showed a significant increase in mean fluorescence intensity in PGE2 compared to the vehicle, *P*=0.0001 (Fig. 4E).

It is well established that the zebrafish embryo does not need an active vascular system for 4–5 days post-fertilization (Gore et al., 2012); however, cardiac cells remain the first line of embryonic cells developed in all vertebrates. Furthermore, heart muscle development and heart rate are the indicators of progressed developmental stages in zebrafish (Kimmel et al., 1995). Thus, we quantified the mean heartbeat of zebrafish larvae at 96 hpf. We captured a 30 s movie of the embryonic heartbeat in all three groups. Video data for non-injected presented in Movie 3A, vehicle injected in Movie 3B and PGE2 injected in Movie 3C. The quantitative data of selected embryos showing a significant increase in the mean of heart beating rate of PGE2 larvae (*n*=15) compared with the vehicle (*n*=12), *P*=0.023 are presented in Fig. 4F. We observed no difference between the vehicle and the non-injected group, quantitative data not presented. The observation of advanced and functional vasculature further suggests that PGE2 promotes early vascular maturation in zebrafish.

### PGE2 induced maturation of vascular anatomy in zebrafish in a time-dependent manner

**Fig. 5.**
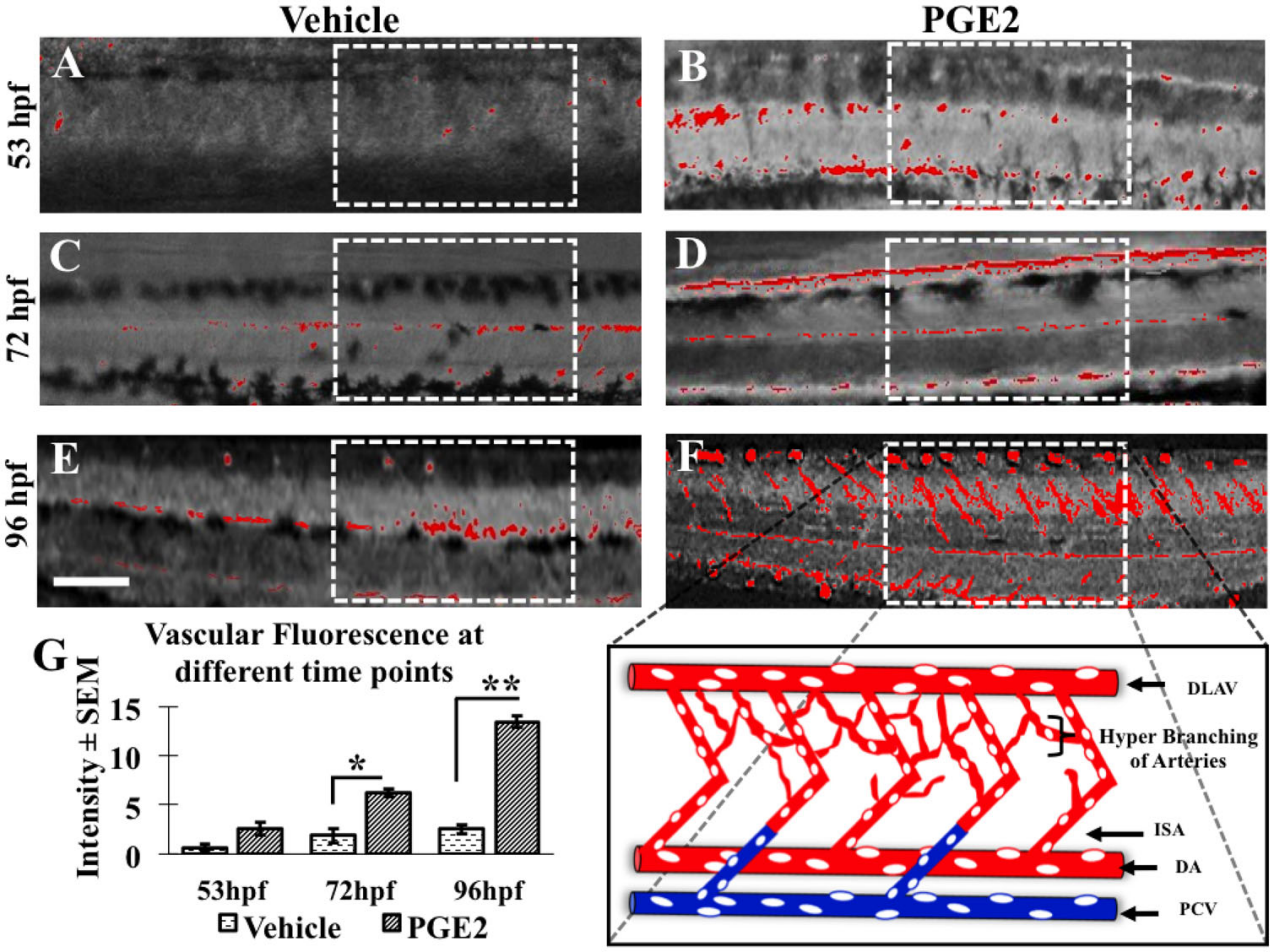
**PGE2 induces angiogenesis in zebrafish in a time-dependent manner.** The dextran dye can trace cell lineage to track vascular development in whole fish. However as the embryo matures, the dye starts to diffuse, which makes it difficult to capture vascular maturation beyond 96 hpf. (A–F) The developing zebrafish trunk vasculature in both vehicle and PGE2 groups from 53 hpf to 96 hpf. The region of interest (ROI) in white dotted boxes shows the difference in vascular fluorescence between groups. (F) In this image, the ROI is expanded to show the dorsal aorta (DA), dorsal longitudinal anastomosing vessel (DLAV), posterior cardinal vein (PCV) and accompanying intersegmental arteries (ISA). (G) We selected a few zebrafish embryos [PGE2 (*n*=9) and vehicle (*n*=9)] in both groups to measure fluorescence intensity with ImageJ. The chart represents the mean fluorescence intensity±s.e.m. An unpaired *t*-test was conducted for each time point and we observed a significant increase in the trunk vasculature fluorescence in the PGE2 compared to the vehicle group at 72 hpf and 96 hpf, respectively. *P=0.007, **P=0.0001. Scale bar: 15 μm.

To establish PGE2-induced vascular maturation in zebrafish, we measured the vascular fluorescence in vehicle and PGE2 groups at different time points (53 hpf, 72 hpf and 96 hpf) (Fig. 5). As the embryonic cell lineage gets differentiated during embryonic development; the fluorescence intensity gets diffused with time and it becomes difficult to minimize background interference and maximize fluorescence *in vivo*. The original fluorescent images were added in the Fig. S1. To quantify only vascular fluorescence, we converted images to grayscale using ImageJ and highlighted region of interest (ROI) with boxes (Fig. 5). We observed a more prominent change in the branching and sprouting of blood vessels during vascular maturation in PGE2 group in a time-dependent manner (Fig. 5A–F). We observed a more developed dorsal aorta (DA), dorsal longitudinal anastomosing vessels (DLAV) and inter-segmental arteries (ISA) in PGE2 larvae (Fig. 5B,D,F) than the vehicle larvae (Fig. 5A,C,E). This observation of vascular anatomy of DA and ISA suggests that PGE2 induces both primary and secondary angiogenesis in zebrafish (data presented with an illustration in Fig. 5F and quantitative data of total vasculature is presented in Fig. 5G). Quantification (*n*=9 for vehicle and *n*=9 for PGE2 at all time-points) showed a significant increase in mean fluorescence intensity in PGE2 embryos compared to the vehicle at 72 hpf, *P*=0.0073, and at 96 hpf, *P*=0.0001 (Fig. 5G). Although the fluorescence of vascular anatomy was high in PGE2 at 53 hpf, the difference was not significant.

### PGE2 regulates angiogenesis and lymphangiogenesis gene expression

**Fig. 6.**
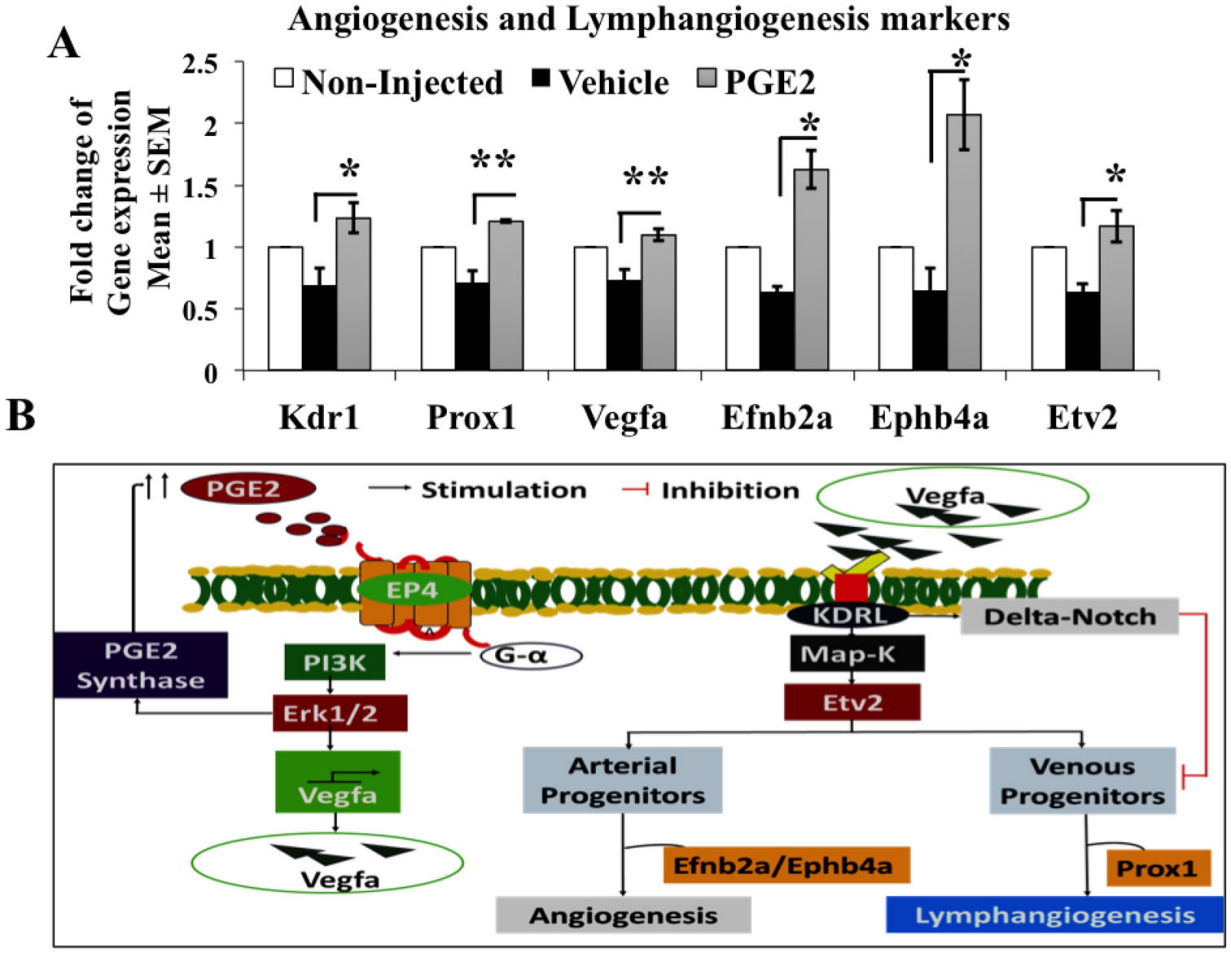
**PGE2 regulates vascular marker mRNA expression in zebrafish.** (A) mRNA qPCR expression analysis of different vascular markers from non-injected, vehicle and PGE2 at 96 hpf. PGE2 shows an upregulation of *vegfa* (1.1-fold), *kdrl1* (1.23-fold), *prox1* (1.21-fold), *etv2* (1.17-fold), *efnb2a* (1.62-fold) and *ephb4a* (2.07-fold) when compared to the vehicle*.* The tested vascular genes were normalized to *actb1* as the control reference gene. For each group, all embryos were pooled before extraction in each replicate. Data represented as the mean of three biological replicates (*n*=3)±s.e.m. Unpaired *t*-test was conducted to compare fold changes for each gene between PGE2 and vehicle groups showing significant differences, *P<0.05, **P<0.01. (B) Possible signaling mechanism of how PGE2 induces angiogenesis in zebrafish.

To examine the expression of vascular genes among the non-injected, vehicle and PGE2 injected groups; we extracted total RNA and performed quantitative RT-PCR using TaqMan gene expression assays. We euthanized embryos at 96 hpf and pooled all embryos in the same group in one tube before RNA extraction. As a result, for gene expression assays, our sample size became *n*=3 for each condition. We observed a drop in gene expression in the vehicle compared to the non-injected group, however that change was not statistically significant. We hypothesize that this difference was observed due to the microinjection, thus, we decided to compare gene expression fold changes in the PGE2-microinjected group to the vehicle-microinjected group. The mRNA fold change results (Fig. 6A) showed a marginal (1.2- to 2.1-fold) change in all gene expressions measured between PGE2 and vehicle groups. We observed a significant increase in NOTCH target genes *efnb2* (*P*=0.003), *ephb4a* (*P*=0.01), angiogenesis markers *vegfa* (*P*=0.02), *kdrl* (*P*=0.04), *etv2* (*P*=0.02) and lymphangiogenesis marker *prox1* (*P*=0.0001) expression in PGE2 group compared to the vehicle (Fig. 6A). Increase in vascular gene expression in PGE2 supports vascular anatomy maturation data.

### COX-2 inhibitor (COX-2I) and EP4 antagonist (EP4A) can abrogate PGE2 induced functions *in vitro*

We used a rat mesenteric lymphatic endothelial cell line (RMLEC) to test the potential of COX-2I NS398 and an EP4A ONO-AE3208 to inhibit PGE2 induced angiogenesis and lymphangiogenesis *in vitro*. Tube formation assay is a surrogate model to test angiogenesis and lymphangiogenesis *in vitro*. The assay was carried out with RMLEC cells seeded on Matrigel with different treatment conditions (Fig. S2). Under native serum-free conditions without any stimulation by virtue of the cells, RMLEC can form very few tubes (a complete network of cells) at 24 h, however in the presence of PGE2, numbers of tubes are significantly increased (*P*=0.0003). Addition of COX-2I and EP4A significantly abrogated PGE2 induced tube formation of RMLEC *P*=0.05 and *P*=0.01, respectively.

## DISCUSSION

It has been established that HSC homeostasis is tightly controlled by PGE2. PGE2 plays a key role in the regulation of embryonic stem cells during embryonic growth and early cell differentiation in zebrafish (Cha et al., 2006). Chemicals that enhance prostaglandin synthesis increase stem cell production, while those that block prostaglandin synthesis decrease stem cell numbers (North et al., 2007). In the adult zebrafish model, PGE2 improved kidney marrow recovery following an irradiation injury and it plays a regulatory role in spleen and bone marrow formation in a murine model (North and Goessling, 2017). The cyclooxygenase-1 (COX-1) enzyme is responsible for PGE2 synthesis in vertebrates and maintains a physiological level of PGE2 to sustain tissue homeostasis. However, overproduction of PGE2 by COX-2 is reported to promote chronic inflammation and breast cancer (Majumder et al., 2018; Lala et al., 2018). Overproduction of PGE2 has specifically been shown to be associated with the breast cancer angiogenesis, lymphangiogenesis and metastasis by upregulation of VEGF-C and VEGF-D (Xin et al., 2012; Majumder et al., 2014).

In the current study, we show that PGE2 induces embryonic growth and upregulates angiogenesis and lymphangiogenesis marker expression. Morphological data show advancement in zebrafish development including an increase in embryonic tail flicks, pigmentation, larval motility and heart rate in presence of PGE2. This suggests that PGE2 might be promoting embryonic development. Here, we observed that PGE2 upregulates *efnb2a*, a NOTCH target gene that is involved in angiogenesis and erythropoiesis. It was shown that PGE2 induces embryonic growth in zebrafish by NOTCH/Wnt upregulation (Hogan et al., 2009). Previously, we have shown that overexpression of PGE2 induces *WNT* and *NOTCH* pathway genes in human breast cancer and PGE2 induces cancer stem-like cells (SLCs), which were abrogated with NOTCH/Wnt and PI3K/Akt and Erk inhibitor treatments (Majumder et al., 2016). These results indicate that PGE2 regulates both embryonic and mature stem cells via EP4, PI3k/Akt and ERK pathways (Majumder et al., 2016; Lala et al., 2018; Cha et al., 2006). Here we found a NOTCH target gene *efnb2a* is upregulated in PGE2 injected embryos, so we need to further investigate other stem cell regulatory pathway genes in zebrafish.

Here we also observed an advancement of zebrafish trunk vascular development, with an increase in intersegmental arteries and lymphatic vessel formation in the PGE2 injected group. This suggests that angiogenesis and lymphangiogenesis occurred together during early embryonic development in zebrafish. We observed a marginal fold change in PGE2 injected group compared to vehicle, suggesting that the effect of externally added PGE2 on vascular gene expression is not so prominent at early embryonic development (96 hpf). Our observation is supported by another study showing that different stages of vertebrate growth are associated with different gene expression profiles (Yang et al., 2013). Therefore, we might observe a noticeable fold change in gene expression if the larvae were to develop into adult zebrafish. Nevertheless, we recorded a significant increase in the percentage of the total vascular fluorescence, with significantly developed vascular anatomy along with the upregulation of angiogenesis genes (*vegfa* and *kdrl)* in the PGE2-injected larvae. Our observation is further supported by another group, which showed that primary angiogenesis sprouts from the dorsal aorta in the trunk of zebrafish are mediated by *vegfc* and *vegfd* interactions with *kdrl* and *flt4*, respectively (Hogan et al., 2009; Bower et al., 2017). Increase in *vegfa* production can also stimulate maturation of vasculature, promote endothelial cells migration and regulate HSC lineages to form red blood cells (Liang et al., 2001). Hence PGE2 induces angiogenesis via upregulation of *vegfa* and *kdrl* in zebrafish*.* We observed a higher expression of lymphangiogenesis marker, *prox1*, in the PGE2 injected group. This might be due to upregulation of *kdrl*, which is a receptor for both *vegf* and *prox1* to promote angiogenesis and lymphangiogenesis. The above findings are supported by studies showing a significant upregulation of *prox1* in PGE2 treated zebrafish (Bower et al., 2017; Shin et al., 2016; Koltowska et al., 2015).

We propose that PGE2 induced upregulation of *vegf* might be via the PI3K/Akt signaling pathway. Here, we observed that PGE2 upregulated tube formation of RMLEC and it was shown previously that PGE2 regulates tube formation in RMLEC by stimulation of PI3K/Akt signaling (Nandi et al., 2017). We reported earlier that COX2/PGE2 induced VEGF-C/D production and tumor associated angiogenesis and lymphangiogenesis was inhibited by blocking EP4/PI3K/Akt signaling in breast cancer models (Xin et al., 2012; Majumder et al., 2014, 2018; Nandi et al., 2017). In zebrafish, the increase in *vegfa/kdrl* interactions upregulates the vascular transcription gene *etv2* via Map-k signaling (Chetty et al., 2017), which in turn modulates the differentiation of arterial and venous expansions in a NOTCH-dependent manner. Overexpression of *vegfa* can repress the venous expansion possibly through the delta-NOTCH pathway, which is independent of *etv2* (Chetty et al., 2017). Here, we observed that PGE2 induces lymphangiogenesis marker *prox1* expression, which facilitates lymphatic differentiation from the venous progenitor stem cells, thus inducing lymphangiogenesis. Our data also suggests that PGE2 induces both angiogenesis and lymphangiogenesis possibly by stimulating the interaction of *vegf* with its cognate receptor *kdrl.* This interaction, in turn, regulates angiogenesis by upregulating downstream NOTCH target genes, *efnb2a* and *ephb4a*. A suggestive schema of possible mechanisms of PGE2 induced vascular maturation in zebrafish is presented in Fig. 6B.

Furthermore, here we demonstrated that COX2-I and EP4A could abrogate PGE2 induced tube formation by RMLEC. High dosages of COX-2 inhibitors are commonly used as anti-cancer and anti-inflammatory drugs to treat human cancers (Harris et al., 2006). Long-term use of COX2-I could cause severe side effects because they block the production of protective prostanoids (Przygodzki et al., 2015) and EP4A was suggested as a better alternative of COX-2I, which spares cardio protective prostanoids (Majumder et al., 2016, 2018). Hence, this study on the regulatory roles of PGE2 during zebrafish vascular development will help us to better understand the adverse side effects observed with PGE2 inhibitors and will play pivotal roles on the path towards identifying new therapeutic targets in breast cancer.

## MATERIALS AND METHODS

### Ethics statement

The Brandon University Animal Care Committee (BUACC) approved the use of zebrafish in this article and we followed the guidelines of the Canadian Council on Animal Care (CCAC).

### Zebrafish maintenance

Zebrafish used in this study were housed in the animal facility at Brandon University, maintained by Dr Christophe LeMoine. The wild-type adult zebrafish (*Danio rerio*) were bred in a plastic tank maintained with Brandon dechlorinated tap water in a 10 h:14 h light dark cycle at 28°C. The fish were fed once daily on Adult Zebrafish Complete Diet (Zeigler, Gardners, PA). The fertilized zebrafish eggs were harvested in hundreds and eggs were raised (one-cell stage) in a glass-plated petri dish filled with E3 embryo medium (in mM: 5 NaCl, 0.17 KCl, 0.33 CaCl, 0.33 MgSO_4_ and 0.00001% Methylene Blue) and kept at 28°C. With the aid of a microinjector (IM 300, Narishige, Long Island, USA) and a pulled 1.0-mm borosilicate glass micropipette (Stutton Instrument, Novato, USA), 1 nl volume of either PGE2 (4 μM) or vehicle (0.13% BSA) along with 1μM of red fluorescent dye (Dextran, Texas Red™, 3000 MW, Lysine Fixable, Thermo Fisher Scientific) were injected into eggs. Following the microinjection, all groups including PGE2-injected, vehicle-injected and the non-injected control were maintained under the same conditions in E3 growth media and incubated at 28°C (LeMoine and Walsh, 2013). Phenotypic changes during embryonic growth of zebrafish were monitored and recorded until 96 hpf with a stereoscopic and a fluorescence microscope.

### Drugs

PGE2 and NS398 were purchased from Cayman (Ann Arbor, USA); ONO-AE3-208 from ONO Pharmaceuticals, Osaka, Japan. Dr Peeyush K. Lala at the University of Western Ontario kindly provided us with all these chemicals.

### Image and video processing

Zebrafish embryos were left submerged in a glass-bottomed petri dish filled with E3 medium during data recording. Images and movies of non-injected and injected (vehicle and PGE2) zebrafish embryos were recorded using a stereoscopic zoom microscope (Nikon SMZ1500) and a fluorescence microscope (Olympus MVX10). Images were further processed and quantified using ImageJ (Simms et al., 2017) and ZFIN (Chávez et al., 2016) software. Movie files were converted and processed using Wondershare Video Converter.

### Zebrafish assays

The fertilized zebrafish eggs were obtained at the one-cell stage, but when we started injecting they reached the two-cell stage. We replicated microinjections for all conditions at least three times. An average of 20 embryos died post injection and the dead embryos were promptly removed. In three experiments the number of embryos used were as follows: in experiment 1, non-injected *n*=30, vehicle injected *n*=40 and PGE2 injected *n*=40; in experiment 2, non-injected *n*=60, vehicle injected *n*=70 and PGE2 injected *n*=70; in experiment 3, non-injected *n*=60, vehicle injected *n*=30 and PGE2 injected *n*=60. The eggs injected with 0.13% BSA served as ‘vehicle’ control of PGE2 treatment, and eggs injected with PGE2 (4 μM) were considered as the ‘PGE2’ treatment group. We also injected Dextran (red fluorescent dye) in both vehicle and PGE2 groups, to trace the phenotypic changes occurring during early development of the embryos. Also, the fluorescent labeling was employed to aid in the selection of embryos that were successfully injected; embryos without any fluorescence in the vehicle and PGE2 groups were excluded from the study. The vehicle group was first injected, followed by the PGE2 group, with a 1-h time gap maintained throughout the growth-monitoring period. Phenotypic changes of zebrafish embryonic growth in the three groups were observed and recorded and developmental stages were compared with Kimmel et al. (1995).

### RNA extraction and gene expression assays

At 96 hpf, zebrafish larvae were euthanized by freezing at −80°C for 2 h and then we added QIAzol lysis reagent (QIAzol^®^, Qiagen) followed by homogenization of the tissue with vigorous vortexing. Then we extracted total RNA using miRNeasy Mini Kit (Qiagen) and synthesized cDNA using high-performance TaqMan mRNA cDNA Reverse Transcription Kit (Life Technologies). Quantitative RT-PCR was done with TaqMan Gene Expression Assays (Life Technologies) using a Rotor-Gene 6000 Real-Time PCR (Corbett Research^®^). Vascular gene expression for *kdrl* (Dr03432884_m1), *vegfa* (Dr03435728_m1), *prox1* (Dr03086822_m1), *efnb2a* (Dr03073975_m1), *ephb4a* (Dr03138278_m1) and *evt2* (Dr03077892_m1) were normalized to the values of *actb1* (Dr03432610_m1) control gene expression by calculating the relative changes between threshold cycle (Ct) of the vascular genes and the control gene (*actb1*) (ΔCT) within each group. The relative fold change in gene expression between the PGE2 and vehicle groups were quantified by calculating ΔΔCt (the ΔCT of treatment group - ΔCT of reference group), followed by fold change of gene expression (2^−ΔΔct^) as described previously (Majumder et al., 2015; Majumder et al., 2016, 2018).

### Tube formation assay

Rat mesenteric lymphatic endothelial cell line (RMLEC) is a spontaneously immortalized LEC isolated from rat mesenteric lymphatic endothelium (Whitehurst et al., 2006), kindly provided by Dr Peeyush K. Lala (University of Western Ontario). RMLEC forms tube-like structure on Matrigel, which is an *in vitro* surrogate of angiogenesis and lymphangiogenesis processes. RMLEC was grown in Dulbecco's Modified Eagle Medium (DMEM) supplemented with 10% FBS, 2 mM glutamine, 50 U/ml penicillin and 50 μg/ml streptomycin, 1 mM sodium pyruvate, and 1 mM nonessential amino acids (all products from Gibco) at 37°C in a humidity maintained CO_2_ incubator. Tube formation assay was carried out with RMLEC cells under different treatment conditions on BD Matrigel™ (BD Biosciences, USA). Matrigel was thawed overnight at 4°C, diluted with cold sterile PBS (Gibco) in 1:1 ratio, and 0.25 ml/well was used to coat 24-well culture plates (VWR, ON) and left at 37°C for 6 h. After polymerization, 40,000–60,000 cells/well, suspended in DMEM medium were added to each well. Under native serum-free condition, very low levels of tube formation occurred at 24 h but PGE2 (20 μM) treatment significantly stimulated tube formation. In a separate experiment to test the involvement of COX-2 and EP4 pathways in PGE2 induced tube formation; we added COX-2I NS398 (20 μM) and EP4A ONO-AE3208 (20 μM) in addition to PGE2. We took 10 to 15 random pictures per well in all experimental conditions using an inverted microscope (Nikon). The numbers of total tubes per unit area were quantified using the ImageJ software as reported earlier (Majumder et al., 2012; Nandi et al., 2017).

### Statistical analysis

Statistical calculations were performed using GraphPad Prism software version 5. Data were presented as mean±standard error of mean (s.e.m.) for each experiment. Unpaired *t*-test was used when comparing the mean of two datasets to estimate two-tailed *P*-value. Statistically relevant differences between means were accepted at *P*<0.05.

## Supplementary Material

Supplementary information
