# Supplementary material for: Prostaglandin E2 promotes embryonic vascular development and maturation in zebrafish
Source: Biol Open. 2019 Apr 15;8(4):bio039768. doi: 10.1242/bio.039768 (PMC6504002; doi:10.1242/bio.039768)
Supplement: Supplementary information [file biolopen-8-039768-s1.pdf]

Supplementary Figures

Supplementary Figure 1

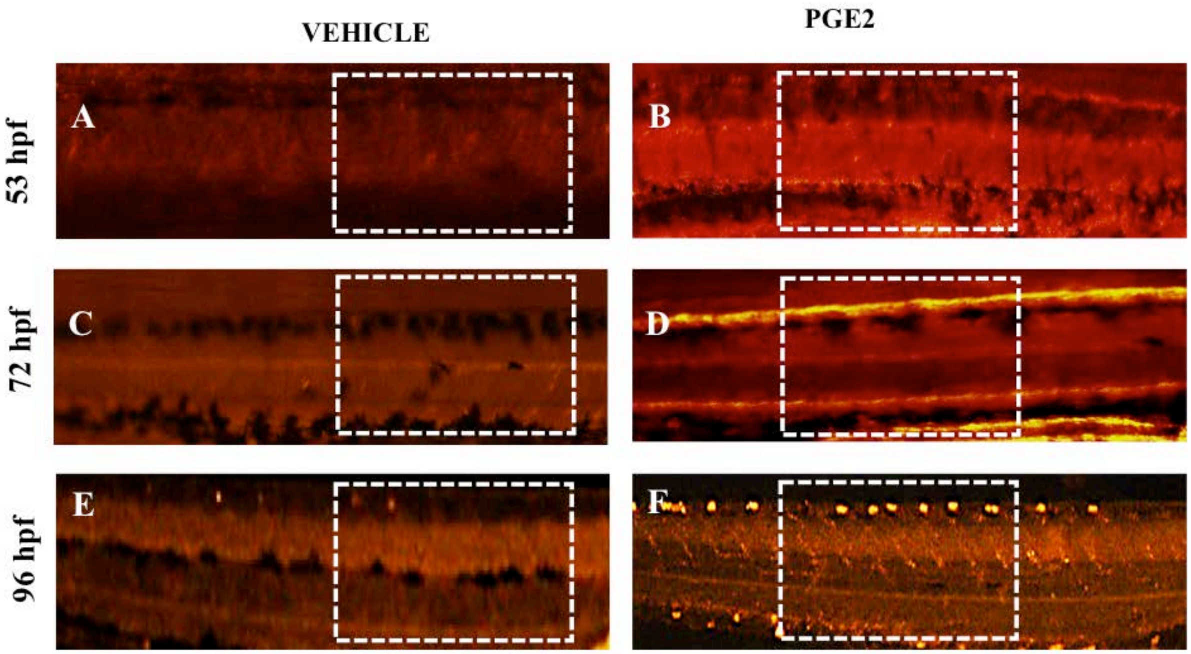

Figure S1. We captured vascular development of hatched zebrafish embryos at different time points 53, 72 and 96hpf with a fluorescent microscope. A region of interest (ROI) for vascular maturation was shown with white dotted rectangle boxes. Vascular development for the vehicle at three-time points are presented in A, C, E and for PGE2 presented in B, D, F.

Supplementary Figure 2

Tube Formation Assay with Rat Mesenteric Lymphatic Endothelial Cells

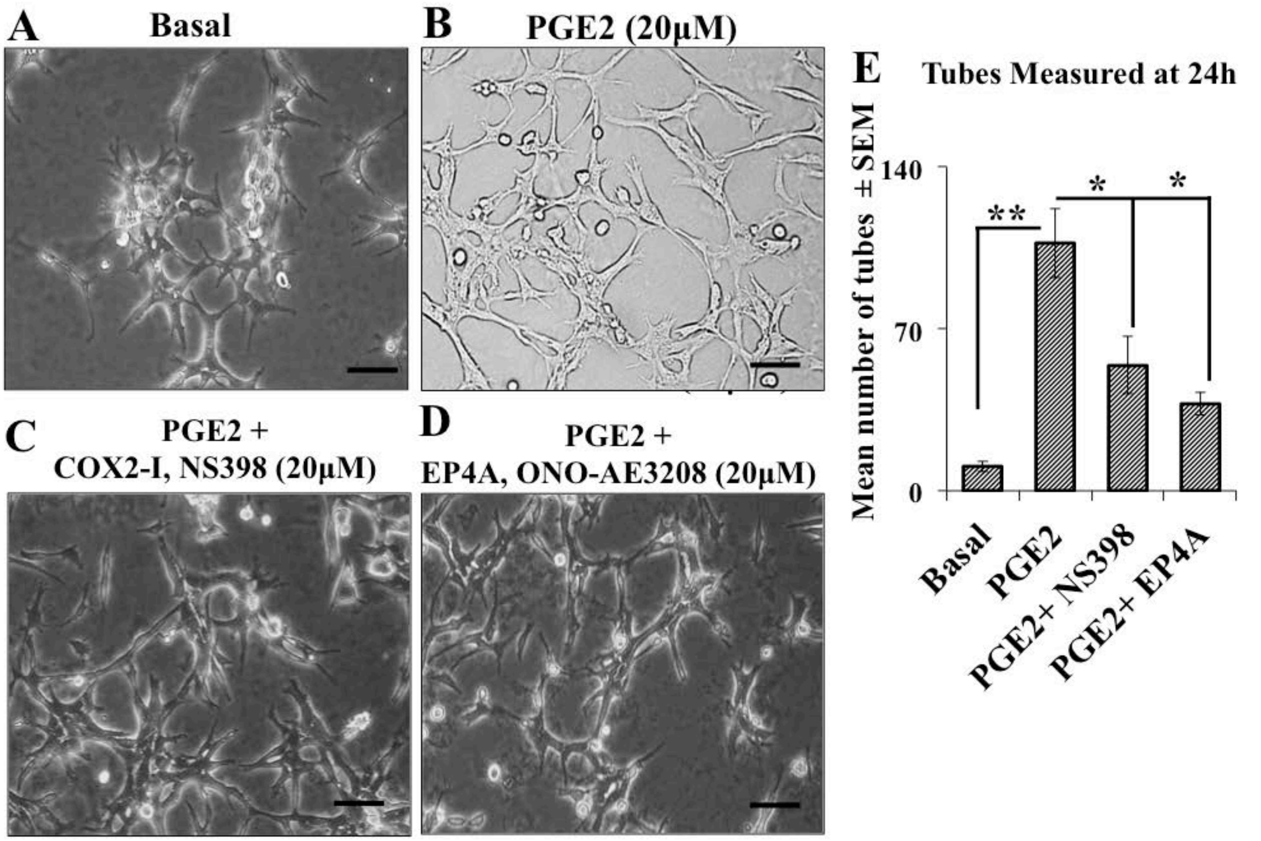

Figure S2. RMLEC forms tubes on Matrigel. (A) In basal media RMLEC forms very few tubes, (B) PGE2 induced tube formation and in presence of COX-2 inhibitor NS-398 (C) and EP4 antagonist ONO-AE3208 (D) PGE2 induced tube formation abrogated. Scale bar represents 200μM. (E) We quantified number of total tubes (complete cell network) at 24h and data presented as the mean of three experiments (n=3) ± SEM. Unpaired t-test showing the increase in tube formation in presence of PGE2 (**P=0.003) and NS398 (*P=0.05) and ONO-AE3208 could significantly block PGE2 induced tube formation.

Supplementary Movies

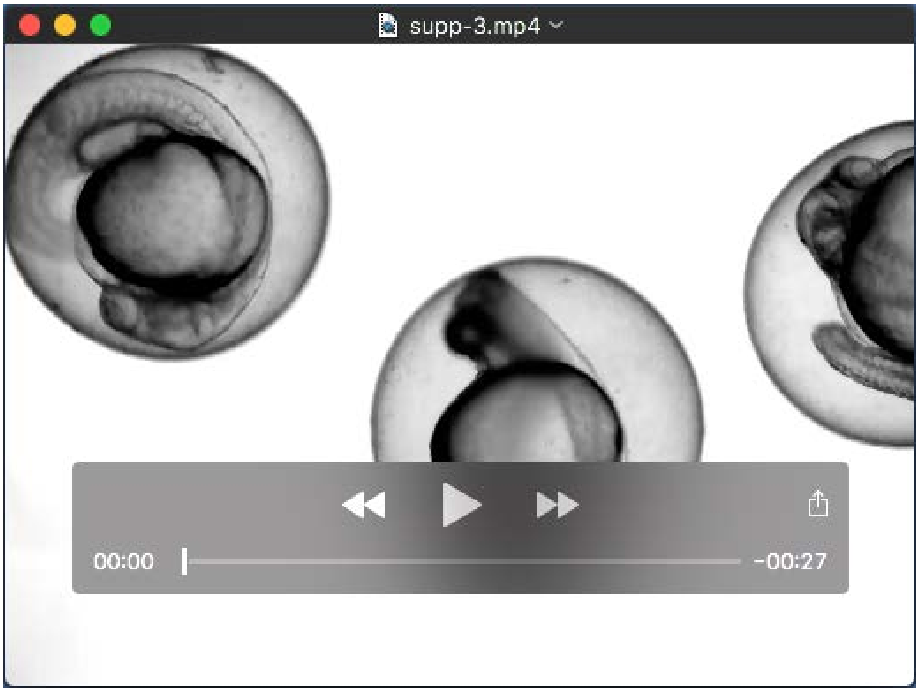

Movie 1A

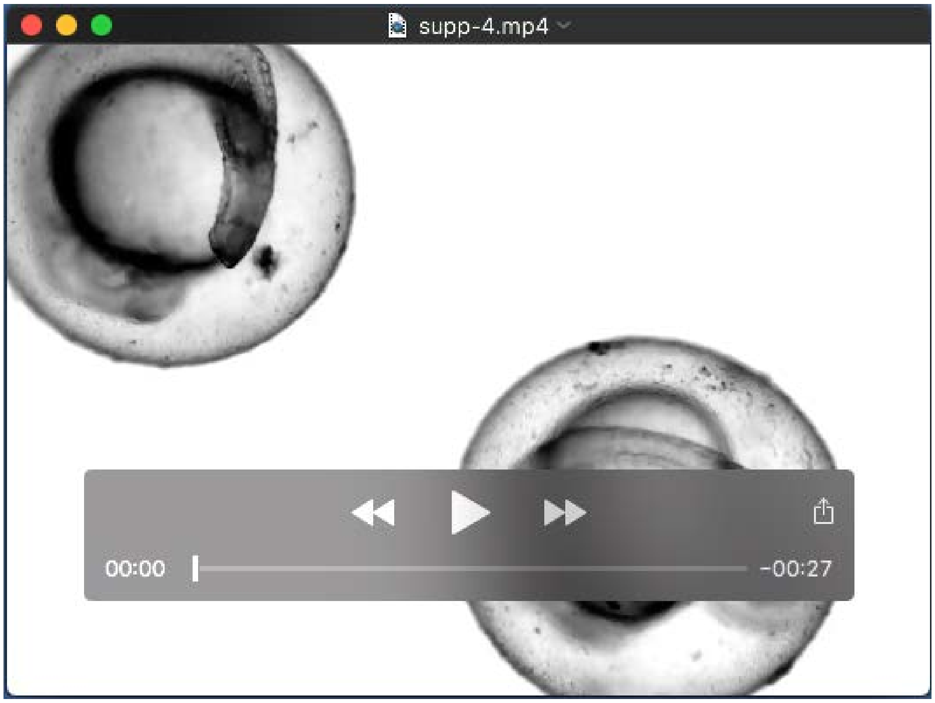

Movie 1B

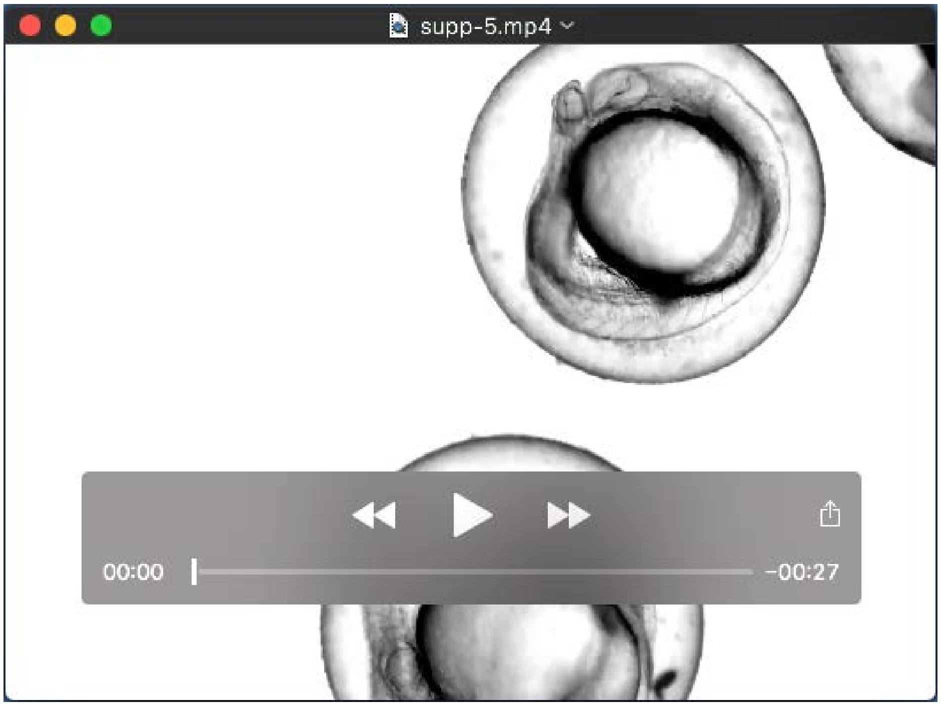

Movie 1C

Movie 1: PGE2 increases embryonic tail-flick in zebrafish at 28hpf. (A-C) Shows a 30-second movie representative of embryos from normal, vehicle and PGE2 at 28hpf. The frequency of the embryonic tail-flicks was observed from three replicates of group. No difference was observed in the frequency of embryonic tail-flick between the normal (movie 1A) and the vehicle (movie 1B). The embryonic tail-flick of the PGE2 (movie 1C) was observed to be more frequent than the vehicle.

Biology Open • Supplementary information

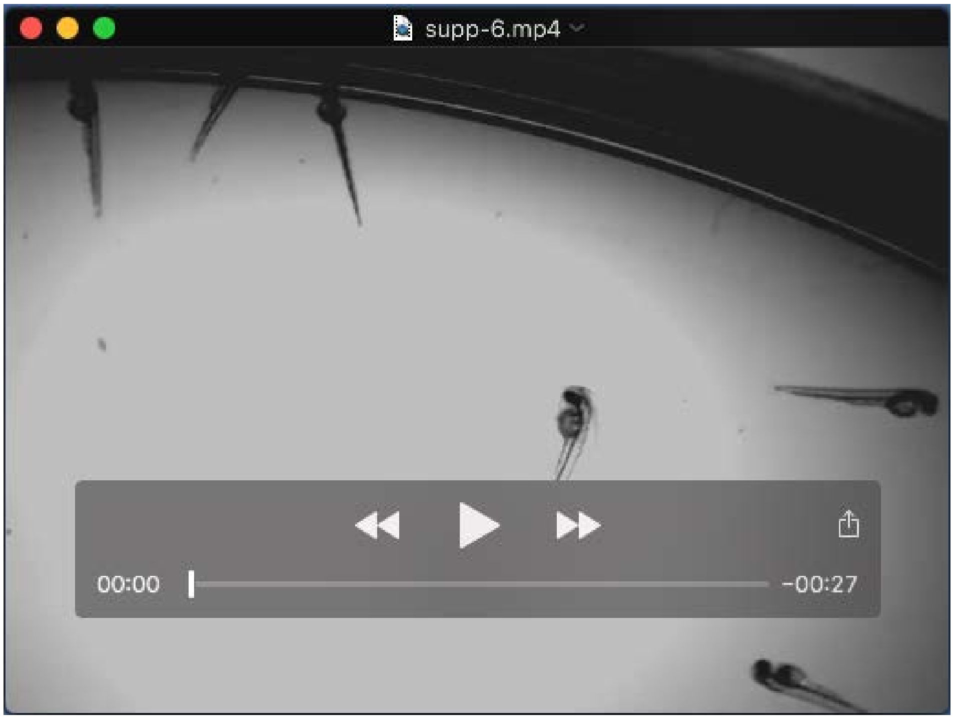

Movie 2A

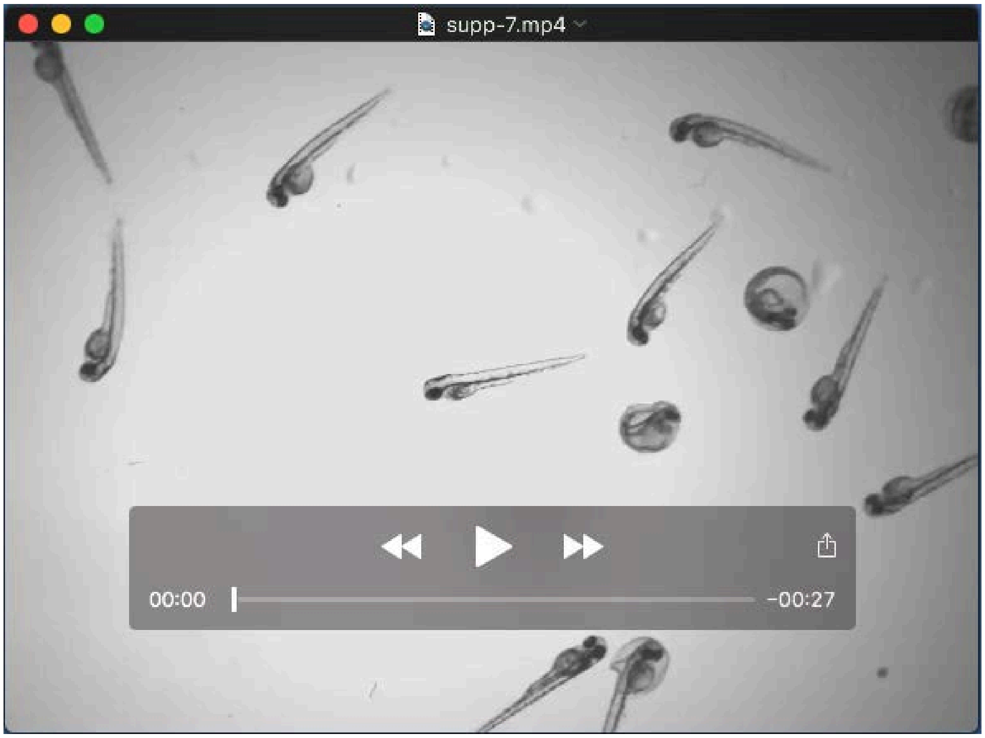

Movie 2B

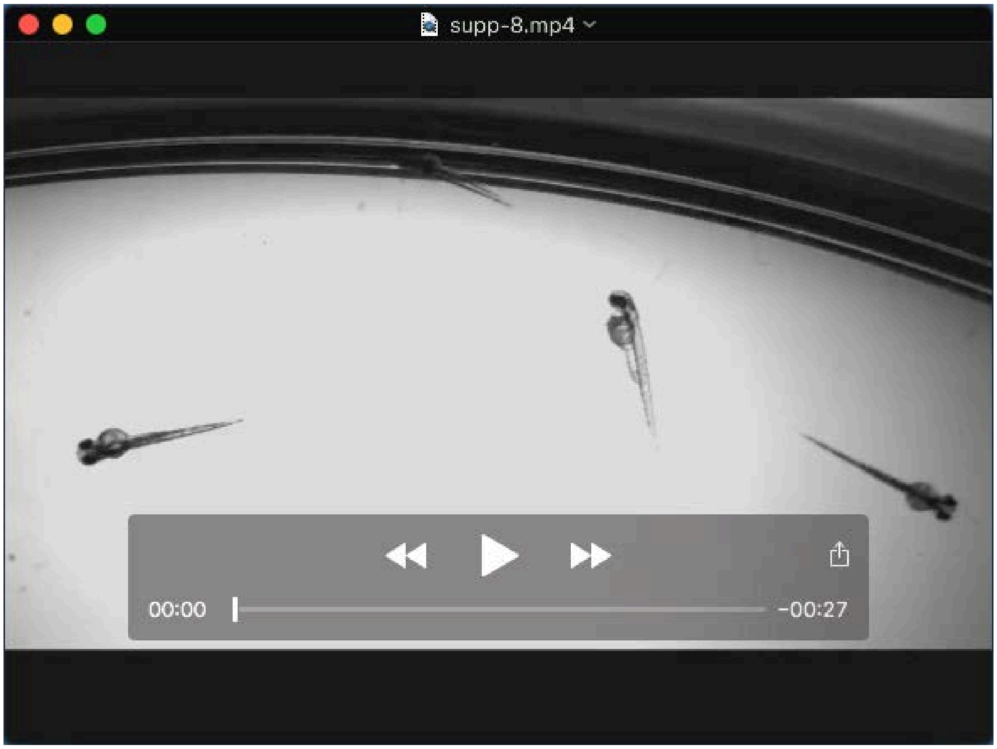

Movie 2C

Movie 2: **PGE2 promotes swimming activity in zebrafish at 50hpf. (A-C)** Shows a 30-second movie of the normal (movie 2A), vehicle (movie 2B) and PGE2 (movie 2C) larvae at 50hpf after hatching. Zebrafish larvae in PGE2 show more active and frequent motility than the vehicle and normal.

Biology Open • Supplementary information

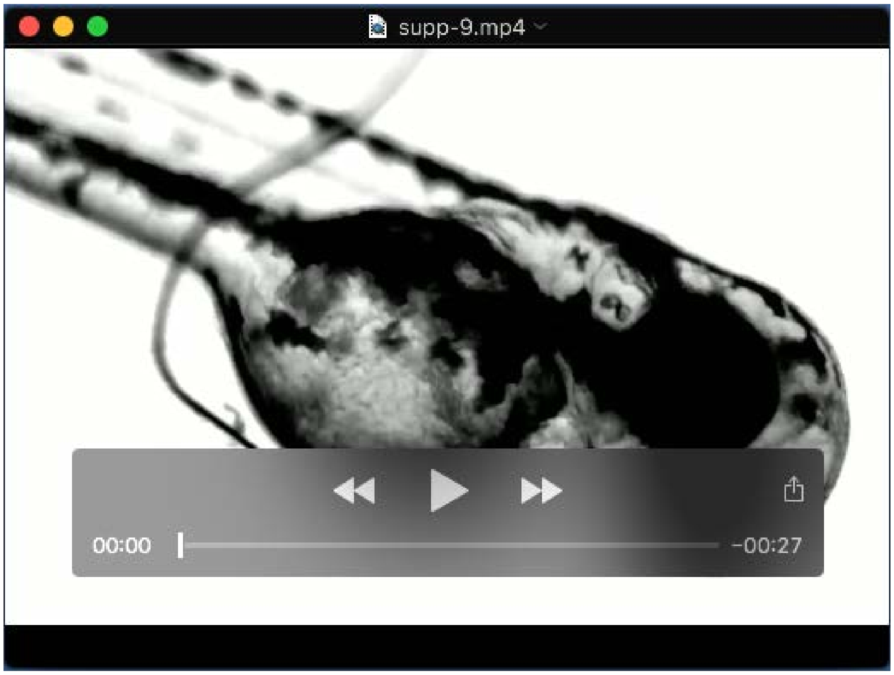

Movie 3A

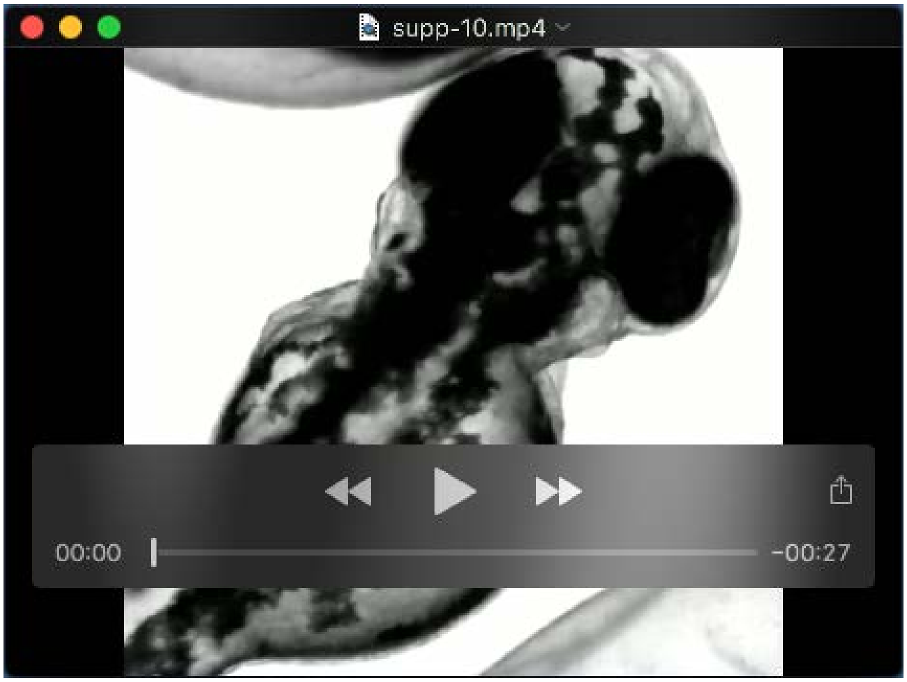

Movie 3B

Biology Open • Supplementary information

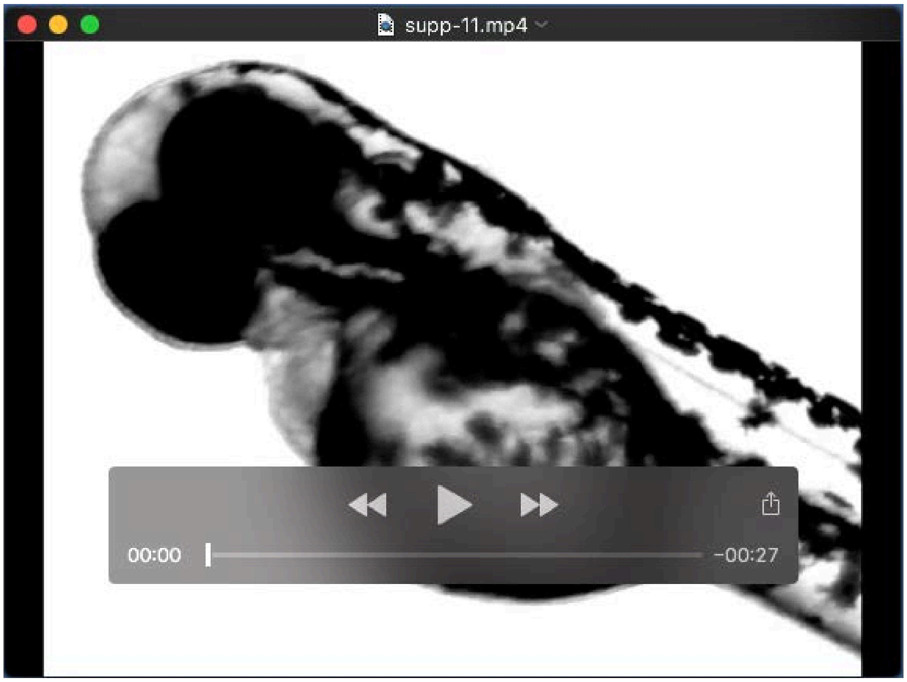

Movie 3C

Movie 3: **PGE2 promotes increased heartrate in zebrafish at 96hpf. (A-C)** Shows a 30-second movie of the normal (movie 3A), vehicle (movie 3B) and PGE2 (movie 3C) larvae at 96hpf. Zebrafish larvae in PGE2 show faster heart beats than the vehicle and the normal.

Biology Open • Supplementary information
